# Successful Adaptation in the Context of Care Environments: Promise and Challenge From a Career in Geropsychology

**DOI:** 10.1093/geroni/igaa057.2087

**Published:** 2020-12-16

**Authors:** Suzanne Meeks

**Affiliations:** University of Louisville, Louisville, Kentucky, United States

## Abstract

My first clinical exposure to older adults was in a psychiatric hospital, to people with life-long severe mental illnesses; I was drawn to their perseverance. The older people we see clinically interact with care environments that may or may not effectively use patients’ personal histories. I have studied the affective experiences of older people in care environments--the mental health system and long-term care--to improve mental health care and well-being. Solutions seem obvious, and difficult. Theory and abundant empirical research tell us that environments can support adaptation through nurturing strengths, offering compensatory tools, and acknowledging losses. My work on depression is a simple example of how this can work in psychotherapy. Yet implementing simple solutions means overcoming barriers of training, resources, and institutional inertia. The promise is understanding age as the dynamic representation of a life span; the challenge is making this understanding work for older people.

